# Association of clinical outcome and imaging endpoints in extensive ischemic stroke—comparing measures of cerebral edema

**DOI:** 10.1007/s00330-024-10694-8

**Published:** 2024-04-16

**Authors:** Vincent Geest, Paul Steffen, Laurens Winkelmeier, Tobias D. Faizy, Christian Heitkamp, Helge Kniep, Lukas Meyer, Kamil Zelenak, Thomalla Götz, Jens Fiehler, Gabriel Broocks

**Affiliations:** 1 Department of Neuroradiology, University Medical Center Hamburg-Eppendorf, Martinistraße 52, 20251 Hamburg, Germany; 2 Department of Radiology, Comenius University’s Jessenius Faculty of Medicine and University Hospital, Martin, Slovakia; 3 Department of Neurology, University Medical Center Hamburg-Eppendorf, Hamburg, Germany

**Keywords:** Net water uptake, Midline shift, Brain edema, Stroke, Ischemia

## Abstract

**Objectives:**

Ischemic edema is associated with worse clinical outcomes, especially in large infarcts. Computed tomography (CT)–based densitometry allows direct quantification of absolute edema volume (EV), which challenges indirect biomarkers like midline shift (MLS). We compared EV and MLS as imaging biomarkers of ischemic edema and predictors of malignant infarction (MI) and very poor clinical outcome (VPCO) in early follow-up CT of patients with large infarcts.

**Materials and methods:**

Patients with anterior circulation stroke, large vessel occlusion, and Alberta Stroke Program Early CT Score (ASPECTS) ≤ 5 were included. VPCO was defined as modified Rankin scale (mRS) ≥ 5 at discharge. MLS and EV were quantified at admission and in follow-up CT 24 h after admission. Correlation was analyzed between MLS, EV, and total infarct volume (TIV). Multivariable logistic regression and receiver operating characteristics curve analyses were performed to compare MLS and EV as predictors of MI and VPCO.

**Results:**

Seventy patients (median TIV 110 mL) were analyzed. EV showed strong correlation to TIV (*r* = 0.91, *p* < 0.001) and good diagnostic accuracy to classify MI (EV AUC 0.74 [95%CI 0.61–0.88] vs. MLS AUC 0.82 [95%CI 0.71–0.94]; *p* = 0.48) and VPCO (EV AUC 0.72 [95%CI 0.60–0.84] vs. MLS AUC 0.69 [95%CI 0.57–0.81]; *p* = 0.5) with no significant difference compared to MLS, which did not correlate with TIV < 110 mL (*r* = 0.17, *p* = 0.33).

**Conclusion:**

EV might serve as an imaging biomarker of ischemic edema in future studies, as it is applicable to infarcts of all volumes and predicts MI and VPCO in patients with large infarcts with the same accuracy as MLS.

**Clinical relevance statement:**

Utilization of edema volume instead of midline shift as an edema parameter would allow differentiation of patients with large and small infarcts based on the extent of edema, with possible advantages in the prediction of treatment effects, complications, and outcome.

**Key Points:**

*• CT densitometry–based absolute edema volume challenges midline shift as current gold standard measure of ischemic edema.*

*• Edema volume predicts malignant infarction and poor clinical outcome in patients with large infarcts with similar accuracy compared to MLS irrespective of the lesion extent.*

*• Edema volume might serve as a reliable quantitative imaging biomarker of ischemic edema in acute stroke triage independent of lesion size.*

## Introduction

Besides necrosis of brain parenchyma, the development of ischemic edema is the second major pathophysiological hallmark of ischemic stroke [1, 2]. Particular attention in the context of ischemic edema is paid to patients with large hemispheric infarcts because ischemic edema can have a major impact on the patient’s outcome by leading to a critical increase of intracranial pressure [3, 4]. Furthermore, in light of the recently published trials that investigated the effect of endovascular thrombectomy in patients with large baseline infarcts, ischemic edema might serve as an important endpoint for treatment effects [5–7]. Stroke imaging therefore requires imaging biomarkers that allow early and accurate quantification of ischemic edema.

The most established imaging method for the quantification of ischemic edema is the measurement of midline shift (MLS) [8–11]. In this approach, absolute volume of edema is indirectly estimated by quantifying its mass effect on the cerebral midline. The strength of this method is its simple and rapid application. However, only edema associated with very large infarcts causes MLS. Edema of smaller territorial infarcts areis also associated with worse outcomes [12], and is usually not reflected by MLS. Moreover, the extent of MLS is determined by other space-relevant factors in addition to edema, such as the volume of subarachnoid space [13–15]. These issues are addressed by the concept of CT densitometry–based measurements of ischemic edema. This technique allows the quantification of the relative water content of an infarct based on its relative hypodensity, which is referred to as net water uptake (NWU) [16, 17], and by reference of NWU to the total volume of the infarct (TIV), it allows direct quantification of the absolute edema volume (EV) [18].

The aim of this study was to compare EV and MLS, determined on early follow-up non-contrast head CT, as imaging biomarkers of ischemic edema and as predictors of edema-induced malignant infarction (MI) and very poor functional outcomes (VPCO), defined as modified Rankin Scale (mRS) 5–6 at discharge. Because MLS can only detect edema of large infarcts, we studied a population of patients with anterior circulation large baseline infarcts, defined as Alberta Stroke Program Early CT Score (ASPECTS) ≤ 5, due to large vessel occlusions anticipated for thrombectomy treatment.

We hypothesized that (1) the accuracy of EV to predict MI and clinical outcome is similar compared to MLS as established gold standard and that (2) EV remains a reliable imaging biomarker for edema quantification in infarcts without apparent MLS.

## Methods

### Study population

For this study, anonymized data from the local stroke registry of the University Medical Center Hamburg-Eppendorf were retrospectively analyzed. The study was conducted in accordance with the ethical guidelines of the local ethics committee (Ärztekammer Hamburg) and in accordance with the Declaration of Helsinki.

All patients admitted between June 2015 and July 2019 that met the following criteria were included: (1) acute ischemic stroke with occlusion of the M1 segment of the middle cerebral artery (MCA) or distal occlusion of the internal carotid artery (ICA); (2) admission multimodal CT protocol with non-enhanced CT (NECT), CT angiography (CTA), and CT perfusion (CTP); (3) an initial ASPECTS of 5 or less in admission NECT rated by a board-certified neuroradiologist; (4) absence of intracranial hemorrhage, preexisting thromboembolic, hemodynamic infarctions, or preexisting significant carotid stenosis on admission CT; (5) documented clinical outcome at discharge assessed using the mRS.

The patients were treated according to current guidelines [19, 20]. This means that patients received early rehabilitation with physiotherapy, speech and language therapy, and occupational therapy within 24 hours of admission as well as anticoagulant medication depending on the risk of hemorrhage and stroke etiology.

### Image acquisition

All CT scans were performed on a 256-slice scanner (Philips iCT 256) with the following imaging parameters: (1) NECT with 120 kV, 280–320 mA, and 5.0-mm slice reconstruction. Filtered back projection with standard kernel was used for image reconstruction at 5-mm-thick contiguous sections, field of view 22 cm, and matrix size 512 × 512; (2) CT angiography with 100–120 kV, 260–300 mA, 1.0-mm slice reconstruction, 5-mm MIP reconstruction with 1-mm increment, 0.6-mm collimation, 0.8 pitch, H20f soft kernel, 80-mL contrast medium, and 50-mL NaCl flush at 4 mL/s; scan starts 6 s after bolus tracking at the level of the ascending aorta. Images were reconstructed at 1.25-mm thickness and 0.625-mm intervals with standard kernel filtered backprojection; (3) CT perfusion with 80 kV, 200–250 mA, 5-mm slice reconstruction (max. 10 mm), slice sampling rate 1.50 s (min. 1.33 s), scan time 45 s (max. 60 s), biphasic injection with 30 mL (max. 40 mL) of highly iodinated contrast medium with 350 mg iodine/mL (max. 400 mg/mL) injected with at least 4 mL/s (max. 6 mL/s) followed by 30-mL NaCl chaser bolus. All datasets were inspected for quality and excluded in case of severe motion artifacts.

### Image analysis

The anonymized CT datasets were processed at the local imaging core lab and segmented manually using commercially available software (Analyze 11.0; Biomedical Imaging Resource, Mayo Clinic, Rochester, MN).

The initial ASPECTS on admission CT and the MLS on admission and follow-up CT were rated by two experienced neuroradiologists separately with subsequent consensus reading. The follow-up CT was screened for secondary parenchymal hemorrhage and for contrast staining in case thrombectomy was performed. Furthermore, volumetric measurements of the hypodense infarct lesions were performed manually on follow-up NECT to derive TIV.

According to an established standardized procedure, NWU was quantified using CT densitometry. This method is based on the proportional relationship between the volume of water uptake and the density reduction of tissue in NECT [16, 17, 21]. First, the infarct lesion was identified as hypodense lesion in NECT. On admission CT, perfusion images (blood volume parameter (CBV) maps at a fixed window between 0 and 6 mL/100 mL) were used to confirm the judgment of NECT hypodensity. To obtain the density, a region of interest (ROI) was placed in the infarct lesion (*D*_ischemic_) and was then mirrored symmetrically within the normal tissue of the contralateral hemisphere (*D*_normal_). Quantitative NWU was calculated based on *D*_ischemic_ and *D*_normal_ according to Eq. 1 [18]:

1 $$\mathrm{Net\;water\;uptake\;}[\mathrm{\%}] =\left(1- \frac{{D}_{{\text{ischemic}}}}{{D}_{{\text{normal}}}}\right)\times 100$$

To derive the volume of ischemic edema, NWU was multiplied with TIV according to Eq. 2 [18]:

2 $$\mathrm{Edema\;Volume\;}\left[{\text{mL}}\right]=\mathrm{Total\;infarct\;Volume\;}\left[{\text{mL}}\right]\times \mathrm{ Net\;water\;uptake\;}[\mathrm{\%}]$$

Malignant middle cerebral artery infarction (MI) was defined based upon imaging and clinical assessment of the patient as infarct lesions with significant space-occupying mass effect with > 1/2 affected middle cerebral artery territory (at follow-up CT imaging targeted 24 h after admission), with imaging signs of herniation (significant midline shift), and/or with clinical signs of herniation (worsening symptoms with decline of consciousness and anisocoria) requiring decompressive hemicraniectomy and/or leading to death due to direct implications of stroke [3, 14, 22–24].

### Statistical analysis

Analyses and data visualization were performed using STATA (version 17.0; Stata Corp., USA, 2021). Baseline characteristics were compared between patients with very poor clinical outcomes (VPCO; mRS > 4 at discharge) and better outcomes (mRS of 0–4 at discharge) (Table 1). Continuous variables were reported as medians and interquartile ranges (IQR), and categorical variables as counts and percentages. Differences between categorical variables and continuous variables were examined using the chi-squared test (*χ*^2^) and Mann–Whitney *U* test, respectively.

**Table 1:** Patient, procedural, and imaging characteristics dichotomized by clinical outcome

	Total	mRS 0–4 at discharge	mRS 5–6 at discharge	*p* value
	*N* = 70	*N* = 29	*N* = 41	
Age (years), median (IQR)	72 (61–81)	68 (57–81)	73 (68–81)	0.170^1^
Female sex, *n* (%)	27 (39%)	7 (24%)	20 (49%)	**0.037**^2^
Admission				
Time from symptom onset to admission (hours), median (IQR)	4 (2–5)	4 (2–5)	4 (2–4)	0.230^1^
NIHSS, median (IQR)	18 (14–20)	16 (13–18)	19 (16–20)	**0.009**^1^
ASPECTS, median (IQR)	4 (3–5)	4 (3–5)	4 (4–5)	0.655^1^
NWU (%), median (IQR)	9 (7–12%)	9 (5–12%)	9 (7–12%)	0.880^1^
MLS (mm), median (IQR)	0 (0–0)	0 (0–0)	0 (0–0)	0.810^1^
Acute therapy				
MT, *n* (%)	46 (66%)	22 (76%)	24 (59%)	0.130^2^
MT TICI 2b-3, *n* (%)	26 (37%)	16 (55%)	10 (24%)	**0.009**^2^
i.v. administration of rtPA, *n* (%)	40 (57%)	18 (62%)	22 (54%)	0.480^2^
Follow-up imaging (24 h)				
TIV (mL), median (IQR)	110 (51–167)	82 (48–112)	128 (84–196)	**0.006**^1^
NWU (%), median (IQR)	24 (16–28%)	18 (14–26%)	25 (21–30%)	**0.021**^1^
EV (mL), median (IQR)	25 (11–43)	15 (9–25)	34 (15–54)	**0.002**^1^
MLS (mm), median (IQR)	2 (0–4)	0 (0–2)	3 (0–5)	**0.004**^1^
Clinical course				
Malignant infarction, *n* (%)	15 (21%)	5 (17%)	10 (24%)	0.470^2^
Hemicraniectomy, *n* (%)	10 (14%)	5 (17%)	5 (12%)	0.550^2^
sICH, *n* (%)	2 (3%)	0 (0%)	2 (5%)	0.230^2^

Characteristics were compared between patients with mRS 0–4 and mRS 5–6 with the use of either the Mann–Whitney *U* test^1^ for continuous variables and chi-square test^2^ for categorical variables

*mRS*, modified Rankin scale; *NIHSS*, National Institutes of Health Stroke Scale; *ASPECTS*, Alberta Stroke Program Early CT Score; *NWU*, net water uptake; *MLS*, midline shift; *MT*, mechanical thrombectomy; *TICI*, thrombolysis in cerebral infarction; *i.v. rtPA*, intravenous recombinant tissue plasminogen activator; *TIV*, total infarct volume; *EV*, edema volume; *sICH*, symptomatic intracerebral hemorrhage; *IQR*, interquartile range

The relationship between the surrogates MLS, NWU, and EV was examined using regression analyses and scatter plots.

Univariable and multivariable logistic regression models were built to test whether MLS and EV were independent predictors of VPCO. The selection of independent variables was based on previous studies [25]. The model for prediction of VPCO included age, gender, time from onset to admission, admission National Institutes of Health Stroke Scale (NIHSS), ASPECTS, intravenous application of recombinant tissue plasminogen activator (rtPA), and successful mechanical thrombectomy (thrombolysis in cerebral infarction (TICI) 2b–3) (see Table 2). The model for prediction of MI included age, admission NIHSS, ASPECTS, and successful mechanical thrombectomy (TICI 2b–3) (see Table 3).

**Table 2:** Univariable and multivariable logistic regression to predict very poor clinical outcome

Independent variables	Dependent variable: very poor outcome (mRS 5–6 at discharge)								
	Univariable logistic regression			Multivariable logistic regression					
				EV model			MLS model		
	OR	95% CI	*p* value	aOR	95% CI	*p* value	aOR	95% CI	*p* value
EV 24 h	1.03	1.00–1.06	0.031	1.03	1.00–1.06	0.031	–	–	–
MLS 24 h	1.29	1.05–1.58	0.017	–	–	–	1.38	1.08–1.78	0.010
Age	1.03	0.99–1.08	0.103	1.04	1.01–1.08	0.018	1.03	0.98–1.09	0.290
Female gender	2.99	1.05–8.53	0.040	4.09	0.95–17.5	0.058	3.05	0.77–12.1	0.113
Time onset admission	0.85	0.66–1.09	0.191	0.97	0.72–1.32	0.865	1.02	0.75–1.40	0.874
NIHSS	1.19	1.04–1.36	0.013	1.17	1.00–1.36	0.044	1.12	0.95–1.30	0.169
ASPECTS	1.00	0.66–1.52	0.988	1.61	0.93–2.78	0.091	2.14	1.10–4.18	0.025
i.v. rtPA	0.71	0.27–1.87	0.484	0.47	0.13–1.76	0.264	0.37	0.10–1.39	0.139
MT TCI 2b	0.26	0.09–0.72	0.010	0.50	0.13–1.84	0.296	0.23	0.06–0.87	0.030

*EV*, edema volume; *MLS*, midline shift; *NIHSS*, National Institutes of Health Stroke Scale; *ASPECTS*, Alberta Stroke Program Early CT Score; *i.v. rtPA*, intravenous recombinant tissue plasminogen activator; *MT*, mechanical thrombectomy; *TICI*, thrombolysis in cerebral infarction

**Table 3:** Univariable and multivariable logistic regression to predict malignant infarction

Independent variables	Dependent variable: malignant infarction								
	Univariable logistic regression			Multivariable logistic regression					
				EV model			MLS model		
	OR	95% CI	*p* value	aOR	95% CI	*p* value	aOR	95% CI	*p* value
24 h EV	1.04	1.01–1.07	0.003	1.05	1.01–1.07	0.028	x	x	x
24 h MLS	1.45	1.16–1.81	0.001	x	x	x	1.65	1.17–2.32	0.004
Age	0.91	0.86–0.97	0.002	0.86	0.79–0.94	0.001	0.87	0.74–0.93	0.002
NIHSS	1.10	0.96–1.27	0.180	1.10	0.89–1.35	0.398	0.97	0.76–1.24	0.808
ASPECTS	0.60	0.37–0.96	0.034	0.49	0.26–0.93	0.073	0.92	0.38–2.22	0.854
MT TICI2b–3	0.35	0.09–1.37	0.132	0.44	0.05–3.49	0.43	0.06	0.00–1.07	0.055

*EV*, edema volume; *MLS*, midline shift; *NIHSS*, National Institutes of Health Stroke Scale; *ASPECTS*, Alberta Stroke Program Early CT Score; *MT*, mechanical thrombectomy; *TICI*, thrombolysis in cerebral infarction

To compare the performance of EV and MLS as predictors of VPCO and MI, receiver operating curve (ROC) analyses of the univariable regression models were performed. Differences between ROC area under the curve (AUC) were examined using the DeLong test [26]. The optimal cutoff point, its specificity, and its sensitivity were empirically estimated using the Liu and Youden method [27]. A statistically significant difference was accepted at a *p* value of < 0.05.

## Results

### Patients

In total, 70 patients met the inclusion criteria. The median age was 72 years (IQR 61 to 81 years) and 27 (39%) patients were women. On admission, the median ASPECTS was 4 points (IQR 3 to 5). Early follow-up CT after 24 h showed a median TIV of 110 mL. A subgroup analysis of the patients without any MLS in early follow-up CT (*n* = 29) revealed a median EV of 14 mL with an interquartile range (Q1, Q3) of 6–26 mL.

In 15 (21%) patients, MI occurred. These patients were significantly younger and had a lower ASPECTS despite shorter time from onset to imaging. Follow-up CT after 24 h showed larger TIV and EV with a correspondingly greater displacement of the midline (see Table 1). Hemicraniectomy was performed in 10 (67%) patients with MI (see Table 4).

**Table 4:** Patient, procedural, and imaging characteristics dichotomized by occurrence of malignant infarction

	Total	Non-malignant infarction	Malignant infarction	*p* value
	*N* = 70	*N* = 55	*N* = 15	
Age (years)	72 (61–81)	75 (65–81)	57 (55–72)	**0.003**^1^
Female gender, *n* (%)	27 (39%)	21 (38%)	6 (40%)	0.900^2^
Admission				
Time from symptom onset to admission (hours)	4 (2–5)	4 (2–5)	3 (2–4)	**0.045**^1^
NIHSS	18 (14–20)	17 (14–20)	18 (16–20)	0.190^1^
ASPECTS	4 (3–5)	5 (4–5)	3 (3–5)	**0.020**^1^
NWU (%)	9% (7–12%)	0 (0–0)	0 (0–0)	0.610^1^
MLS (mm)	0 (0–0)	0 (0–0)	0 (0–2)	0.045^1^
Acute therapy				
MT, *n* (%)	46 (66%)	35 (64%)	11 (73%)	0.480^2^
MT with TICI2b-3, *n* (%)	26 (37%)	23 (42%)	3 (20%)	0.120^2^
i.v. administration of rtPA, *n* (%)	40 (57%)	30 (55%)	10 (67%)	0.400^2^
Follow-up imaging (24 hours after admission)				
TIV (mL)	110 (51–167)	87 (48–150)	167 (118–262)	** < 0.001**^1^
NWU (%)	24% (16–28%)	0 (0–0)	0 (0–0)	0.530^1^
EV (mL)	25 (11–43)	20 (9–40)	42 (18–68)	**0.003**^1^
MLS (mm)	2 (0–4)	0 (0–3)	4 (3–11)	** < 0.001**^1^
Clinical course and outcome				
Hemicraniectomy, *n* (%)	10 (14%)	0 (0%)	10 (67%)	0.470^2^
sICH, *n* (%)	2 (3%)	1 (2%)	1 (7%)	0.320^2^
mRS at discharge	5 (4–5)	5 (4–5)	5 (4–6)	0.240^1^
Very poor outcome, *n* (%) (mRS ≥ 5 at discharge)	41 (59%)	31 (56%)	10 (67%)	0.470^2^

Characteristics were compared between patients with malignant infarction and those without with the use of either the ^1^Mann-Whitney *U* test for continuous variables or the ^2^chi-square test for categorical variables

*mRS*, modified Rankin scale; *NIHSS*, National Institutes of Health Stroke Scale; *ASPECTS*, Alberta Stroke Program Early CT Score; *NWU*, net water uptake; *MLS*, midline shift; *MT*, mechanical thrombectomy; *TICI*, thrombolysis in cerebral infarction; *i.v. rtPA*, intravenous recombinant tissue plasminogen activator; *TIV*, total infarct volume; *EV*, edema volume; *sICH*, symptomatic intracerebral hemorrhage; *IQR*, interquartile range

Forty-one patients (59%) exhibited VPCO, and they were significantly older, were more often female, had higher admission NIHSS, and were less likely to be successfully recanalized. The follow-up CT after 24 h revealed larger TIV, higher NWU, larger EV, and greater MLS (see Table 1).

### Correlation analysis

**Fig. 1:**
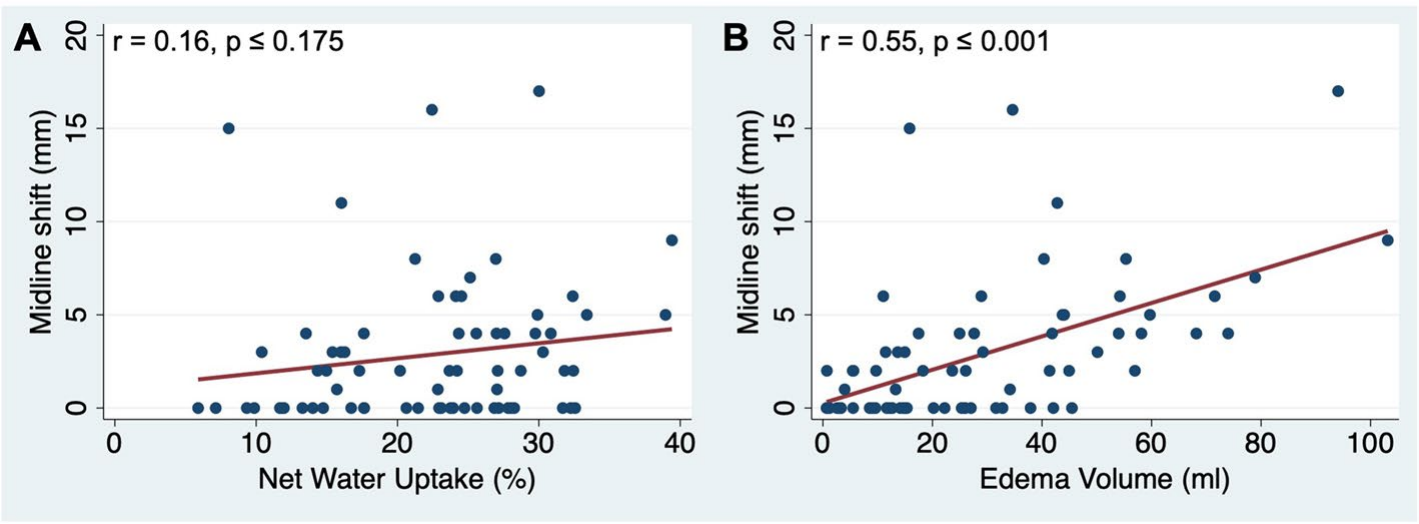
Association between midline shift (MLS) and (**A**) net water uptake (NWU) and (**B**) edema volume (EV) in 24-h follow-up CT with results of correlation analysis. EV was significantly correlated with MLS, while NWU was not

**Fig. 2:**
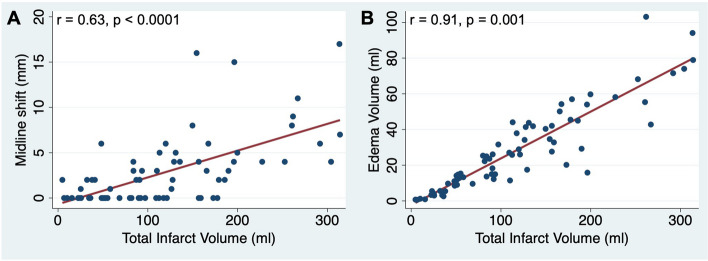
Association between total infarct volume and (**A**) midline shift and (**B**) edema volume in 24-h follow-up CT with results of correlation analysis. EV showed stronger correlation with TIV than MLS

**Fig. 3:**
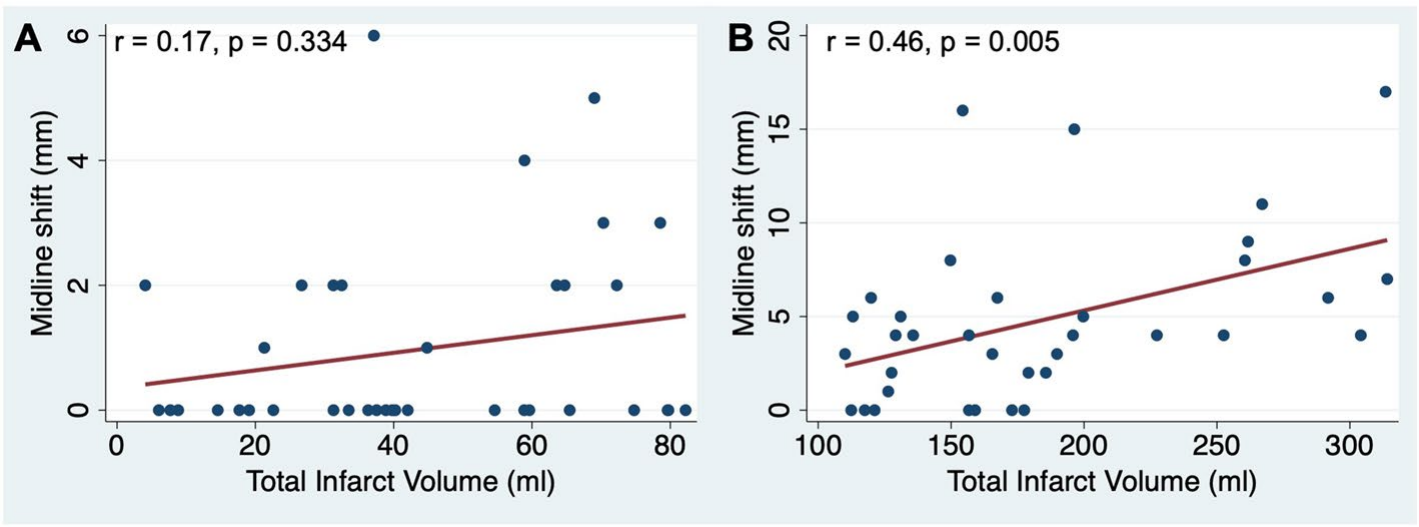
Association between midline shift (MLS) and total infarct volume (TIV) (**A**) < median (110 mL) and (**B**) > median (110 mL) with regression line and results of correlations analysis. MLS showed no significant correlation to infarcts with TIV < 110 mL

Correlation analysis showed no correlation between NWU and MLS (*r* = 0.16, *p* = 0.175). EV and MLS, however, were significantly correlated (*r* = 0.55, *p* < 0.001) (see Fig. 1). Both MLS and EV were correlated to TIV, with EV having a stronger correlation (*r* = 0.91, *p* < 0.001 vs. 0.67, *p* < 0.001) (see Fig. 2). A subgroup analysis of patients with smaller TIV (< median TIV of 110 mL) revealed no correlation for MLS (*r* = 0.17, *p* = 0.334) while the correlation coefficient of EV remained similar (*r* = 0.89, *p* < 0.01) (see Fig. 3).

A significant correlation was also observed between both edema parameters and the clinical endpoints, with similar strength for VPCO (*r* = 0.34, *p* < 0.01 vs. 0.31, *p* < 0.01) and a stronger correlation between MLS and MI (*r* = 0.54, *p* < 0.001 vs. 0.39,* p* < 0.001).

### Prediction of malignant infarction

In the respective models, MLS (adjusted odds ratio (aOR) 1.65; 95% CI 1.17–2.32; *p* = 0.004) and EV (aOR 1.05; 95% CI 1.01–1.07; *p* = 0.028) were both independently associated with greater odds of MI (see Table 3). Besides these, only age was independently associated with MI, decreasing the odds of MI by the factor 0.86 with each year of life. In the EV model, age and ASPECTS showed independent associations.

**Fig. 4:**
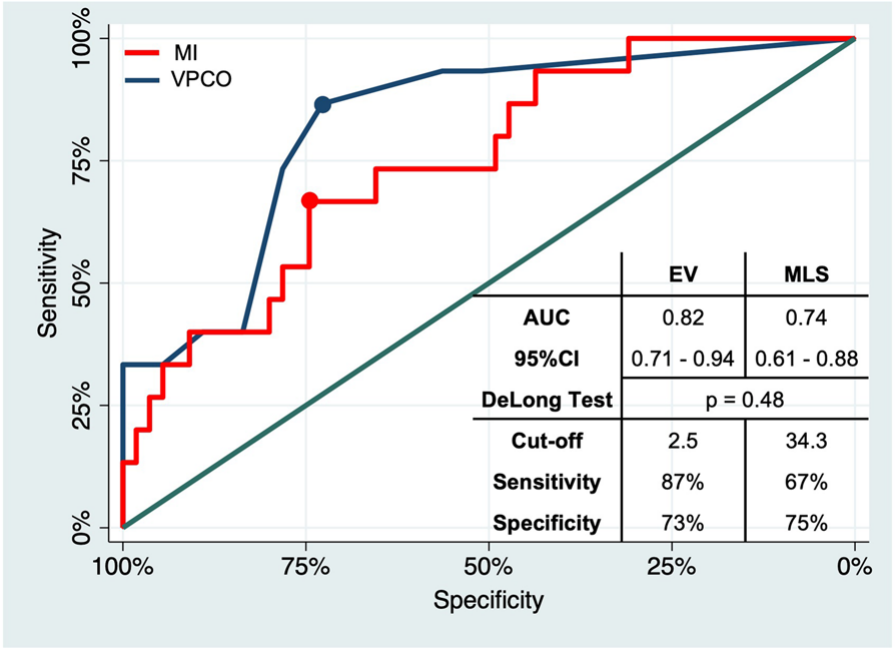
ROC curves of univariate logistic regression with malignant infarction (MI) as a dependent variable and (*blue curve*) midline shift (MLS) or (*red curve*) edema volume (EV) as an independent variable. In addition, AUC with 95% CI, result of the DeLong test, values of the optimal threshold, and its sensitivity and specificity are presented. There was no significant difference between the diagnostic accuracy of EV and MLS to classify MI

Univariate ROC analysis revealed superior discriminatory power of MLS (AUC 0.82; 95% CI 0.71–0.94) compared to EV (AUC 0.74; 95% CI 0.61–0.88), but without significance according to the DeLong test (*p* = 0.304). The optimal thresholds with highest sensitivity and specificity were 28.3 mL EV (sensitivity 63%, specificity 76%) and 2.5 mm MLS (sensitivity 73%, specificity 62%) (see Fig. 4).

### Prediction of very poor clinical outcome

MLS (aOR 1.38; 95% confidence interval (CI), 1.08–1.78; *p* = 0.01) and EV (aOR 1.03; 95% CI, 1.00–1.06; *p* = 0.02) were both independently associated with greater odds of VPCO. Beyond that, age and admission NIHSS were independently associated with VPCO in the EV model, while admission ASPECTS and mechanical thrombectomy with successful reperfusion (TICI 2b–3) were independently associated with VPCO in the MLS model.

**Fig. 5:**
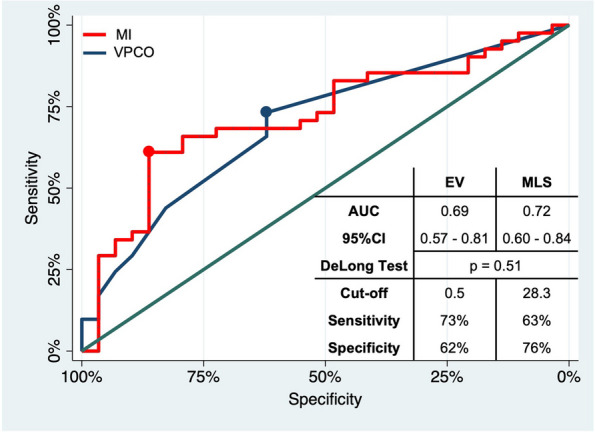
ROC curves of univariate logistic regression with very poor clinical outcome (VPCO) as a dependent variable and (*blue curve*) midline shift (MLS) or (*red curve*) edema volume (EV) as an independent variable. In addition, AUC with 95% CI, result of the DeLong test, and values of the optimal threshold and its sensitivity and specificity are presented. There was no significant difference between the diagnostic accuracy of EV and MLS to classify VPCO

Univariate ROC analysis revealed slightly higher discriminatory power of EV (AUC 0.72; 95% CI 0.60–0.84) compared to MLS (AUC 0.70; 95% CI 0.58–0.82), again with no significant difference according to the DeLong test (*p* = 0.696). The optimal thresholds with highest sensitivity and specificity were 28.3 mL EV (sensitivity 63%, specificity 76%) and 0.5 mm MLS (sensitivity 73%, specificity 62%) (see Fig. 5).

## Discussion

The concept of densitometry provided EV as a new imaging parameter for the quantification of ischemic edema. We aimed to investigate the correlation between EV and the established biomarker MLS, and assessed whether EV may improve the prediction of edema-related VPCO and MI on early follow-up NECT of patients with large baseline infarcts. We found that (1) EV and MLS showed a similar diagnostic accuracy to classify VPCO and MI in large infarcts, and (2) EV has the advantage that it can also quantify and differentiate edema without MLS. EV may serve as a reliable surrogate for outcome prediction of patients with large baseline infarcts likely to develop extensive edema formation over time.

A recently published study by Ng et al also aimed to compare volumetric and densitometric edema quantification. In contrast to our study, Ng et al chose NWU as a densitometric parameter to challenge MLS. Because of lack of association with MLS as well as clinical outcome, Ng et al concluded that NWU should not be used as an edema biomarker in follow-up imaging of post thrombectomy patients. The main reason given for the lack of associations was the confounding of NWU measurements by contrast enhancement and hemorrhagic transformation [28]. Apart from methodological errors that may limit the association between NWU and clinical outcomes, testing the association between NWU and MLS may intrinsically not be plausible. NWU is a measure of the relative edema proportion without any information about the edema’s spatial extent, and therefore independent from spatial parameters such as MLS per se (see Fig. 1A) [16, 17]. EV may expand this concept as it combines the direct and precise character of NWU and spatial information [18]. In line with this, EV showed a good degree of correlation to MLS (see Fig. 1B). Similar results were reported for the correlation of DWI-based swelling volume (MRI correlate to EV) and MLS [11]. The limitation of the correlation between MLS and EV can be explained by the fact that the effect of edema on cerebral midline depends not only on the volume of edema, but also on other factors such as the volume of the residual subarachnoid spaces [13–15]. Thus, MLS reflects edema indirectly in the context of other factors of intracranial space while EV quantifies edema directly and independently of other factors.

Another major difference of EV and MLS is their applicability outside of very large infarcts (see Table 5). Even in our cohort of patients with large baseline infarcts, MLS showed a significant correlation only with infarct volumes above the 50th percentile of 110 mL (see Fig. 3). Consequently, MLS cannot reliably be used for edema quantification in infarcts < 110 mL, despite these edemas also being associated with worse functional outcomes [12]. In contrast, EV showed a similar and good correlation with all infarct volumes determined in this cohort (see Fig. 2B), which is explained by the inclusion of infarct volumes in the calculation of EV. Accordingly, EV has the advantage to quantify edemas within small and large infarcts with similarly high accuracy.

**Table 5:**
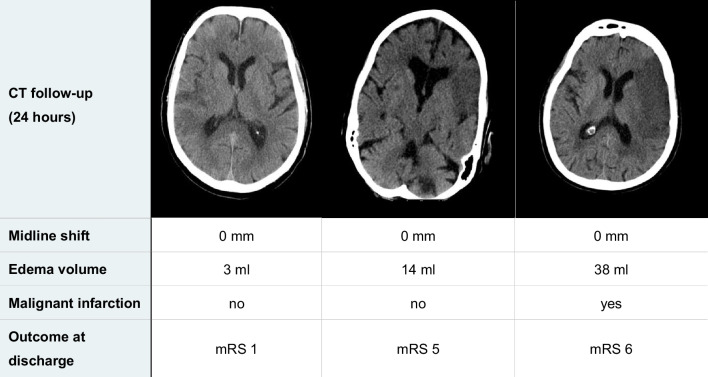
Examples of patients without midline shift but significant differences in edema volume and outcome

Shown are representative axial images of 24-h follow-up head CT with measures of MLS and EV and the clinical endpoint occurrence of malignant infarction and clinical outcome at discharge assessed using modified Rankin scale. Left: Small infarct in the posterior right MCA territory. Middle: Moderate infarct in the anterior and lateral left MCA territory. Right: Large infarct of the left MCA territory

This implies that EV, in contrast to MLS, has a high discriminatory power in differentiating edemas of any size. EV could therefore be a useful endpoint for studies that investigate the effects of edema development on functional outcomes, like CT-based large core trials as the recently published TENSION [29] and the ongoing TESLA (NCT03805308).

After this comparison of EV and MLS in terms of their ability to detect ischemic edema, the question about differences in prediction of edema-associated clinical endpoints remains.

Despite our cohort of patients having large infarcts, where MLS is established, EV showed a good discrimination power for VPCO slightly superior to that of MLS, with no significant difference (AUC 0.72 vs. 0.69, *p* = 0.51; see Fig. 5). These results are in line with previous studies—Nawabi et al, who postulated elevated EV in early follow-up CT as an indicator of futile recanalization, reported an AUC of 0.74 for discrimination of poor outcome by EV (poor outcome was defined here as mRS 4–5, but in the context of smaller infarcts) [30]. Regarding MLS, Ostwaldt et al published an AUC of 0.68 for discrimination of poor outcome in their MRI-based comparison of mass effect biomarkers (poor outcome was defined here as mRS 3–6 in the context of smaller infarcts) [11]. Our analysis of the optimal thresholds showed that the good discrimination power of EV is based on a high specificity, compensating for a lower sensitivity compared to MLS. This was surprising because we expected higher sensitivity for EV due to its ability to also quantify edemas that are not detectable by MLS after 24 h, but do have an impact on outcome due to their progression over time (see Table 5). In fact, patients with no MLS after 24 h but VPCO at admission were rare. It can be assumed that the advantage of EV is more relevant in an even earlier phase of edema development, in which most edemas are still so small that they not lead to MLS. However, EV was beneficial in detecting patients with extensive stroke, who did not suffer very poor outcome. Conceivable examples are patients having a shift of cerebral midline after 24 h despite relatively small edema volume because of an infarct located close to the midline or small subarachnoid spaces.

There was also no significant difference between EV and MLS in the early classification of MI after 24 h (AUC 82 vs. 0.74, *p* = 0.48; see Fig. 4), though MLS tended to be advantageous. Although numerous studies have described MLS as the gold standard among imaging parameters and as diagnostic criterion of MI, to our knowledge, this is the first study that determined the diagnostic accuracy of MLS and EV as predictors of MI. According to the analysis of the optimal thresholds, the tendential advantage was driven by a higher sensitivity of MLS. As MI describes a condition in which the edema leads to an increase in intracranial pressure by exceeding reserve spaces, it is plausible that the higher sensitivity of MLS is based on the fact that MLS, compared to EV, takes reserve spaces into account [14]. MLS can thus correctly classify patients who develop a malignant infarct despite a comparatively low edema volume due to low residual subarachnoid volumes (e.g., due to young age), whereas EV classifies these patients false-negative because it only reflects the edema’s spatial extent.

In summary, we showed that EV not only offers a solution for accurate edema quantification in infarcts of any size, including those without MLS, but also could replace the gold standard, MLS, as an early predictor of VPCO and MI in patients with large infarcts with a diagnostic accuracy that was not significantly different. Advantages of EV compared to MLS are a very accurate quantification of edema’s absolute volume leading to a higher specificity for clinical endpoints. MLS, on the other hand, had higher sensitivity and tended to be advantageous in early prediction of malignant infarction by taking reserve spaces into account. To achieve the most accurate classification of MI and VPCO in patients with large infarcts, EV and MLS should be combined with easy to determine, more sensitive MLS as a screening parameter and more elaborate, more specific EV as a confirmatory parameter. As this study focuses on large infarctions allowing a good comparison of edema-related endpoints, it may serve as a proof-of-concept for further investigations analyzing the value of EV as an imaging biomarker particularly to compare treatment effects.

### Limitations

Our study has all limitations that come along with a single center retrospective study design, including a relatively low number of patients due to strict inclusion criteria. Additionally, limitations include restriction of the study to patients with large infarcts due to the limitations of MLS, and follow-up CT set at a specific time point, with development of ischemic edema being a dynamic process.

Second, hemorrhage within the infarct, e.g., after reperfusion, is a known contraindication for the use of densitometry because of falsification of density measurements. This excludes patients at particularly high risk for VPCO.

Third, other volumetric edema parameters besides MLS, for instance the ratio of hemisphere volumes, were not analyzed.

Fourth, data on comorbidities, medications, and blood findings were not available, limiting our multivariable regression models. Still, with regard to the end points of this study and consistent results from previous studies [12], we are confident that our multivariable regression results are reasonable. The main analyses (comparison of the diagnostic accuracy of MLS versus EV as predictors of VPCO and MI, as well as the correlation analyses of MLS and EV to NWU TIV) are univariate and therefore not affected by missing comorbidities, medications, or blood findings.

Fifth, another limitation is the lack of data on long-term functional outcomes. However, this study focuses on the early post-stroke phase, which is not only of great importance for the planning of post-acute therapy, but also contributes significantly to the long-term outcome [31]. Therefore, we believe that this study leads to reasonable conclusions. Nevertheless, an analysis of the edema parameters in relation to the long-term outcome would be an interesting approach for further studies in this field.

Sixth, the topography of infarcts was not differentiated in this study, as it is not in any of the current large core trials. This is another limitation of our multivariable regression models for VPCO as it has been shown that different ASPECTS regions also contribute differently to functional outcomes [32]. However, the magnitude and significance of the difference, especially between large core infarcts, remain uncertain as indicated by a study in which cumulative ASPECTS was not inferior to location-specific ASPECTS in outcome prediction [33]. As noted under point four, our main analysis is not affected by this.

## Conclusion

In this study of acute stroke patients presenting with large baseline infarcts, we found that EV may serve as an accurate and direct quantitative imaging biomarker for the assessment of ischemic brain edema in infarcts of any size. EV may reliably predict malignant infarction in patients with large baseline infarcts who are at a specific risk of poor clinical outcomes. Further research should investigate the correlation between EV and treatment effects in this subgroup of patients.

## Data Availability

The data that support the findings of this study are available from the corresponding author upon reasonable request.

## Abbreviations

aOR: Adjusted odds ratio
ASPECTS: Alberta Stroke Program Early CT Score
AUC: Area under the curve
CI: Confidence interval
EV: Edema volume
IQR: Interquartile range
MCA: Middle cerebral artery
MI: Malignant infarction
MLS: Midline shift
mRS: Modified Rankin scale
MT: Mechanical thrombectomy
NECT: Non-enhanced computed tomography
NIHSS: National Institutes of Health Stroke Scale
NWU: Net water uptake
ROC: Receiver operating curve
rtPA: Recombinant tissue plasminogen activator
TICI: Thrombolysis in cerebral infarction
TIV: Total infarct volume
VPCO: Very poor functional outcome

## Declarations

### Guarantor

The scientific guarantor of this publication is Gabriel Broocks.

### Statistics and biometry

Statistical analyses of this manuscript were performed by the authors without external advice.

### Study subjects or cohorts overlap

No overlap.

### Methodology

- retrospective
- observational
- performed at one institution
